# Atomic-Scale Design of High-Performance Pt-Based Electrocatalysts for Oxygen Reduction Reaction

**DOI:** 10.3389/fchem.2021.753604

**Published:** 2021-09-16

**Authors:** Jie Ying

**Affiliations:** School of Chemical Engineering and Technology, Sun Yat-sen University, Zhuhai, China

**Keywords:** Pt-based nanomaterials, oxygen reduction reaction, novel nanostructures, important effects, electrocatalytic design

## Abstract

Fuel cells are regarded as one of the most promising energy conversion devices because of their high energy density and zero emission. Development of high-performance Pt-based electrocatalysts for the oxygen reduction reaction (ORR) is vital to the commercial application of these fuel cell devices. Herein, we review the most significant breakthroughs in the development of high-performance Pt-based ORR electrocatalysts in the past decade. Novel and preferred nanostructures, including biaxially strained core–shell nanoplates, ultrafine jagged nanowires, nanocages with subnanometer-thick walls and nanoframes with three-dimensional surfaces, for excellent performance in ORR are emphasized. Important effects of strain, particle proximity, and surface morphology are fully discussed. The remaining changes and prospective research directions are also proposed.

## Introduction

Fuel cells, as clean and effective energy conversion devices that can directly convert the chemical energy of fuels and oxidants to electricity, are highly demanded to meet the urgent requirement of vehicles and exploitation of environmentally sustainable energies (Chong et al., 2018; Pivovar, 2019). The oxidation reaction of fuels at the anode and the oxygen reduction reaction (ORR) at the cathode are the reactions involved in fuel cells (Chen et al., 2019; Bian et al., 2020). Both anode and cathode reactions need catalysts to lower their electrochemical overpotential for high-voltage output (Zhou et al., 2016; Jiao et al., 2021). So far, scarce and expensive Pt-based catalysts are currently the only choice of catalysts in practical fuel cells (Wei et al., 2018; Sievers et al., 2021). In comparison with the fast rate of anodic oxidation reaction, the sluggish kinetics in cathodic ORR is the major hindrance, which demands much higher loading of Pt to achieve a desirable performance in fuel cells (Ying et al., 2014a; Ying et al., 2018a; Xiao et al., 2019). Therefore, it is vitally important to enhance the intrinsic activity of Pt-based electrocatalysts for ORR by reducing the Pt loading without compromising fuel cell performance.

During the past decades, the design and synthesis of Pt-based electrocatalysts for ORR have undergone a rapid development (Ying et al., 2016; Ma et al., 2020), including underpotential deposition (UPD) techniques (Brankovic et al., 2001), transition metal alloying (Greeley et al., 2009), ordered intermetallic structure constructing (Wang et al., 2013), nanoframe building (Niu et al., 2016), jagged nanowire synthesis (Li et al., 2016), and single atom exploiting (Liu et al., 2017). It has now generated plenty of synthesis strategies and novel nanostructures with increasing activity and stability, such as ultrafine jagged Pt nanowires (Li et al., 2016) and PtPb/Pt core/shell nanoplates (Bu et al., 2016a). Moreover, the mass activity of Pt-based nanostructures has been drastically increased, reaching the peak of 13.6 A/mg_pt_ at 0.9 V (Li et al., 2016), which is almost 31 times greater than the U.S. Department of Energy (DOE)’s 2020 target of 0.44 A/mg_pt_ (Capdevila-Cortada, 2019). Currently, the field of synthesis of high-efficiency Pt-based ORR electrocatalysts is experiencing a prosperous development with increasing achievements. It is necessary to provide a brief overview of this type of advanced material in a timely manner for the understanding of the deep reason on the atomic scale for existing high-performance Pt-based electrocatalysts.

In this minireview, we first present salient examples of Pt-based electrocatalysts with novel and preferred nanostructures for excellent performance in ORR. Then, the important but unfamiliar effects, such as strain, particle proximity, and surface morphology, will be thoroughly discussed. Finally, key scientific problems and prospective research directions are also proposed. We focus only on research studies where there have been significant breakthroughs in the developments of Pt-based electrocatalysts in the past decade and the deep reason why those novel nanostructures and important effects could reach the outstanding performance based on currently advanced technologies and theoretical studies.

## Novel Nanostructures for Superior ORR Performance

In the past several decades, great efforts have been focused on the development of the design and synthesis of novel nanostructures of Pt-based catalysts for improving their catalytic activity and stability (Ying et al., 2017; Xiao et al., 2021a; Xiao et al., 2021b). Many research studies have also indicated that the catalytic properties of Pt-based catalysts are highly correlated with their structure, meaning that the catalytic properties of Pt-based catalysts could be manipulated by altering their nanostructures (Zhang et al., 2017; Xu et al., 2020; Dong et al., 2021). With the development of nanotechnology, various Pt-based nanostructures, such as nanoparticles, nanowires, nanotubes, and nanocages, have been synthesized in parallel with significantly enhanced electrochemical performance (Ying et al., 2014b; Sheng et al., 2017; Liu et al., 2019). In this section, emerging Pt-based electrocatalysts with unique nanostructures, including biaxially strained core–shell nanoplates, ultrafine jagged nanowires, nanocages with subnanometer-thick walls and nanoframes with three-dimensional surfaces, are presented.

With regard to biaxially strained core–shell nanoplates, Pt-based core–shell nanostructures have emerged as a promising paradigm to meet required activity targets (Kim et al., 2019; Tao et al., 2020; Li et al., 2021). The most efficient nanostructures for increasing the activity of catalysts for the ORR on the basis of Pt loading have been PtM (M regarded as Ni, Co, or other metals) alloy nanoparticles with a Pt skin (Wang et al., 2014; Luo et al., 2018a; Zhu et al., 2021). However, the poor electrocatalytic stability in long-term durability has been the major problem impeding its practical application due to the formation of a nonuniform Pt-skin shell (Bian et al., 2015). Therefore, it is highly desired to synthesize an ordered metal core with a uniform Pt-skin layer for acquiring both high activity and high stability.

**FIGURE 1:**
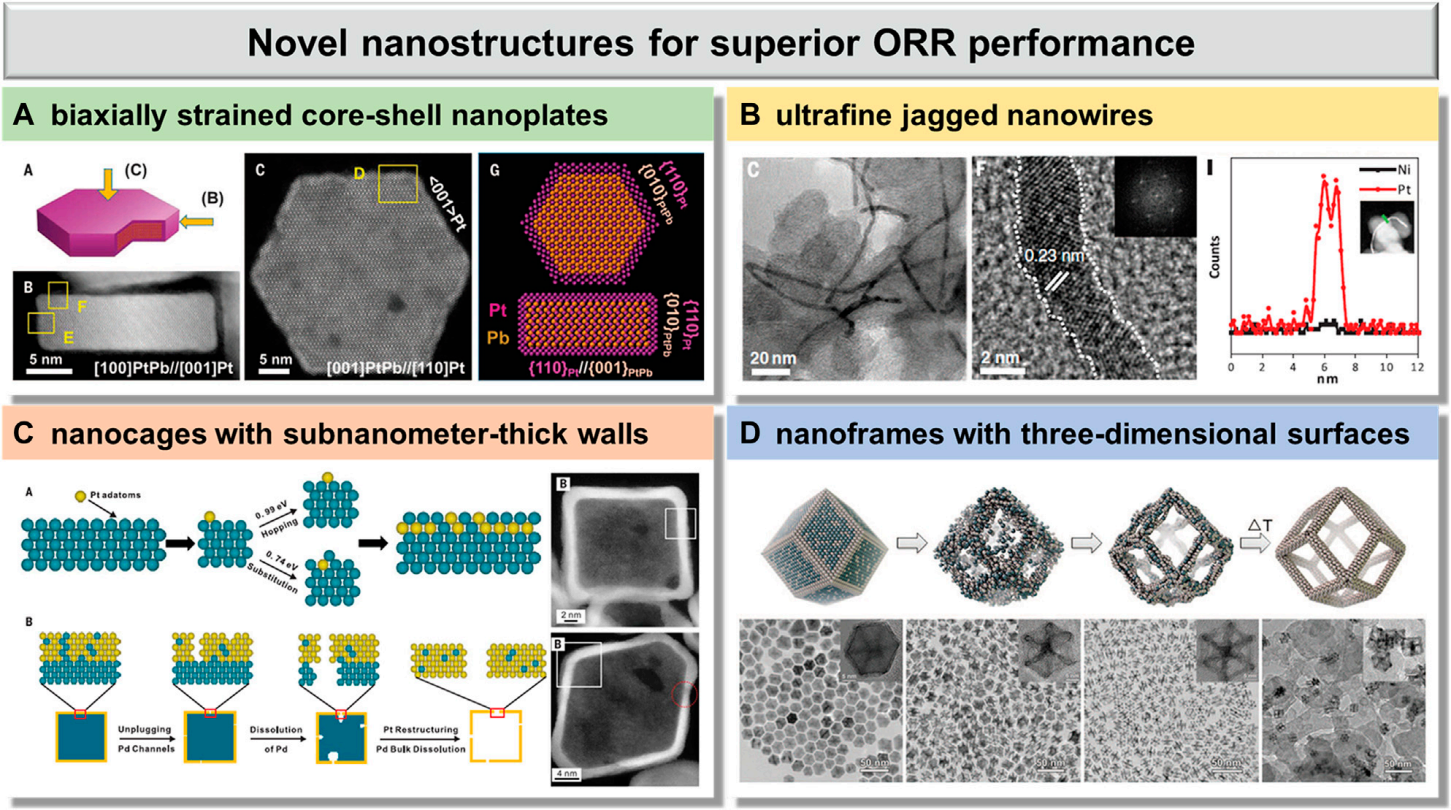
Emerging Pt-based electrocatalysts with novel nanostructures for superior ORR performance: **(A)** biaxially strained core–shell nanoplates, reproduced with permission from ref. 20; **(B)** ultrafine jagged nanowires, reproduced with permission from ref. 18; **(C)** nanocages with subnanometer-thick walls, reproduced with permission from ref. 44; and **(D)** nanoframes with three-dimensional surfaces, reproduced with permission from ref. 48.

A class of highly uniform PtPb/Pt core/shell nanoplates was reported by Huang et al. *via* a one-pot solution synthesis (Figure 1A) (Bu et al., 2016a). The as-synthesized products are hexagonal nanoplates with monodisperse edge length, and a core–shell structure, where the PtPb core is covered with a Pt edge layer. In the nanoplates, the top Pt layers were fully connected to the PtPb core, exhibiting an 11% compressive strain and a 7.5% tensile strain. This unique biaxially strained PtPb/Pt core/shell nanoplate showed high specific and mass activities of 7.8 mA/cm^-2^ and 4.3 A/mg^-1^_pt_ at 0.9 V versus the reversible hydrogen electrode (RHE), which are 33.9 and 27.1 times greater than those of the commercial Pt/C, respectively. They also exhibited outstanding durability with negligible activity decline, unconspicuous structure, and composition changes after 50,000 cycles. The excellent ORR performance can be attributed to their unique biaxial strain, which would be beneficial to optimizing the Pt-O bond on the Pt surface and decreasing the poison effect.

With regard to ultrafine jagged nanowires, Pt-based one-dimensional (1D) nanostructures, such as nanowires, nanorods, and nanotubes, have received tremendous attention all over the world, as they may solve many of the inherent catalytic problems and enhance electrocatalytic activity toward ORR due to their uniquely anisotropic structure, which possesses high aspect ratios, fewer lattice boundaries, high electron transport characteristics, and smooth and low energy facets (Ying et al., 2018b; Huo et al., 2019; Tian et al., 2019). Although the enhancements on electrocatalytic performance observed with Pt-based nanowires are promising, a continuing challenge has been to develop even more highly active and durable catalysts.

Ultrafine jagged Pt nanowires were reported by Duan et al. *via* the selective dealloying approach (Figure 1B) (Li et al., 2016). The jagged Pt nanowires exhibited a very high electrochemically active surface area (ECSA) up to 118 m^2^/g_Pt_, which is much higher than the previous reported highest value of about 70 m^2^/g_Pt_. Notably, the mass activity of the jagged Pt nanowires is up to 13.6 A/mg_Pt_ at 0.9 V, which is 52.3 times that of the state-of-the-art commercial Pt/C catalyst. Moreover, the ultrafine jagged Pt nanowires showed superior durability, experiencing only ∼7% loss of the ECSA, 12% loss of mass activity, and little change in the overall morphology, size, and jagged surface after 6,000 cycles. The ultrahigh ORR activity here is ascribed to the structural feature of the highly stressed and undercoordinated rhombus-rich surface in these jagged nanowires.

With regard to nanocages with subnanometer-thick walls, due to the high surface-to-volume ratio and large void space, hollow structured Pt-based electrocatalysts, such as nanocages, hollow nanospheres, and nanoboxes, have recently received enormous attention for their enhancement of activity in ORR (He et al., 2016; Sun et al., 2017; Asset et al., 2018). In general, the formation of Pt-based hollow nanostructures was evolved from as-synthesized Pt-based alloy core–shell structures by selectively dealloying to remove the core of other active metals.

PtPd nanocages with subnanometer-thick walls and controllable facets were reported by Xia et al. *via* a combination of chemical deposition and etching (Figure 1C) (Zhang et al., 2015). The density functional theory (DFT) calculations revealed that the energy barrier (0.74 eV) of substitution of a Pt adatom into the Pd surface is higher than the energy barrier (0.99 eV) of diffusion of Pt adatoms across the Pd surface. This means that some Pd atoms would enter into the Pt shell during the Pt deposition, rather than the formation of a perfect Pt shell. As a consequence, PtPd cubic and octahedral nanocages with subnanometer-thick walls were obtained by using Pd nanoscale cubes and octahedra as templates, respectively. The mass activity of octahedral nanocages (0.75 Amg^-1^_Pt_) at 0.9 V versus the RHE is over 5 times greater than that of the commercial Pt/C catalyst (0.14 Amg^-1^_Pt_), which is attributed to the ultrathin wall thickness with a bimetallic alloyed structure. Moreover, the ORR mass activity of octahedral nanocages displayed a loss of only 36% after 10,000 cycles, which still show 3.4-fold enhancement compared with the pristine commercial Pt/C catalyst. The catalytic system here could be further optimized before it can compete with the more active system based on Pt-based alloys.

With regard to nanoframes with three-dimensional surfaces, as mentioned above, hollow nanostructures of Pt-based materials have unique physical and chemical properties that differ from many other types of nanostructures. Among the reported hollow nanostructures, nanoframes with totally open three-dimensional configuration have roused ever-increasing interest in the field of eletrocatalysis (Becknell et al., 2017; Chen et al., 2018; Qin et al., 2020). Since catalytic properties of materials can be effectively adjusted by precise control of the nanostructure, it is meaningful to understand in depth the structural evolution of the formation of nanoframes, which could provide guidelines to the precise control of this unique nanostructure for further enhanced catalytic performance.

Highly crystalline Pt_3_Ni nanoframes with three-dimensional (3D) accessible surfaces were reported by Yang et al. *via* spontaneous dissolution of Ni atoms in air (Figure 1D) (Chen et al., 2014). This spontaneous erosion can occur because the surface Ni atoms of PtNi_3_ polyhedra were oxidized by the dissolved oxygen and formed soluble metal complexes with oleylamine ligands, leading to a higher Ni dissolution rate. The specific activity of Pt_3_Ni nanoframes at 0.95 V versus the RHE is >16-fold enhancement compared with the commercial Pt/C, which can be attributed to the formation of a Pt-skin–terminated (111)-like surface structure with a thickness of at least two Pt monolayers. The synergy between high specific activity and the three-dimensional open structures of Pt_3_Ni nanofrmes with both the internal and external accessible surfaces enabled a 22-fold improvement in mass activity compared with the commercial Pt/C. Furthermore, the Pt_3_Ni nanoframes showed extraordinary durability and structure stability, where the activity loss can be negligible and the frame structure remained intact after 10,000 potential cycles between 0.6 and 1.0 V. The excellent durability is attributed to the weaker oxygen binding strength from the electronic structure of the Pt-skin surface, which leads to a lower coverage of oxygenated intermediates, decreasing the probability of Pt dissolution.

## Important Effects for Tuning ORR Performance

For Pt-based nanostructures, the general effects, such as shape, morphology, structure, and catalyst composition, on electrocatalytic activity and stability have been widely studied (Li et al., 2017; Zhang et al., 2019; Kodama et al., 2021). Optimizing the catalyst shape, morphology, structure, and composition is vital to achieving electrocatalysts with superior catalytic activity and stability (Huang et al., 2021; Shen et al., 2021). In addition to these normal effects for Pt-based materials on ORR performance, some in-depth and critical but unfamiliar effects have been investigated recently and are worthy of being highlighted. In this section, these emerging effects, including strain, particle proximity, and surface morphology, will be thoroughly discussed.

Effect of strain. The ultimate goal in catalytic design is to have accurate synthetic control of the material properties. Lattice strain, including compressive strain and tensile strain, based on the atomic arrangement of surface atoms, can change the surface electronic structure by altering the distances between surface atoms and, in turn, catalytic performance and could thus be used to gain precise control of the material properties (Asano et al., 2016; Luo and Guo, 2017; Xia and Guo, 2019). For Pt-based ORR electrocatalysts, previous studies have indicated that the change of only 1% lattice strain can lead to the shift of the 5d-band center of Pt by ∼0.1 eV, resulting in distinct strengthening or weakening of the bonding of reaction intermediates to the surface (Hammer and Nørskov, 2000). Thus, it is vital to fully understand strain effects from theory to achieve a reactivity–strain relationship that can provide guidelines for tuning ORR performance.

**FIGURE 2:**
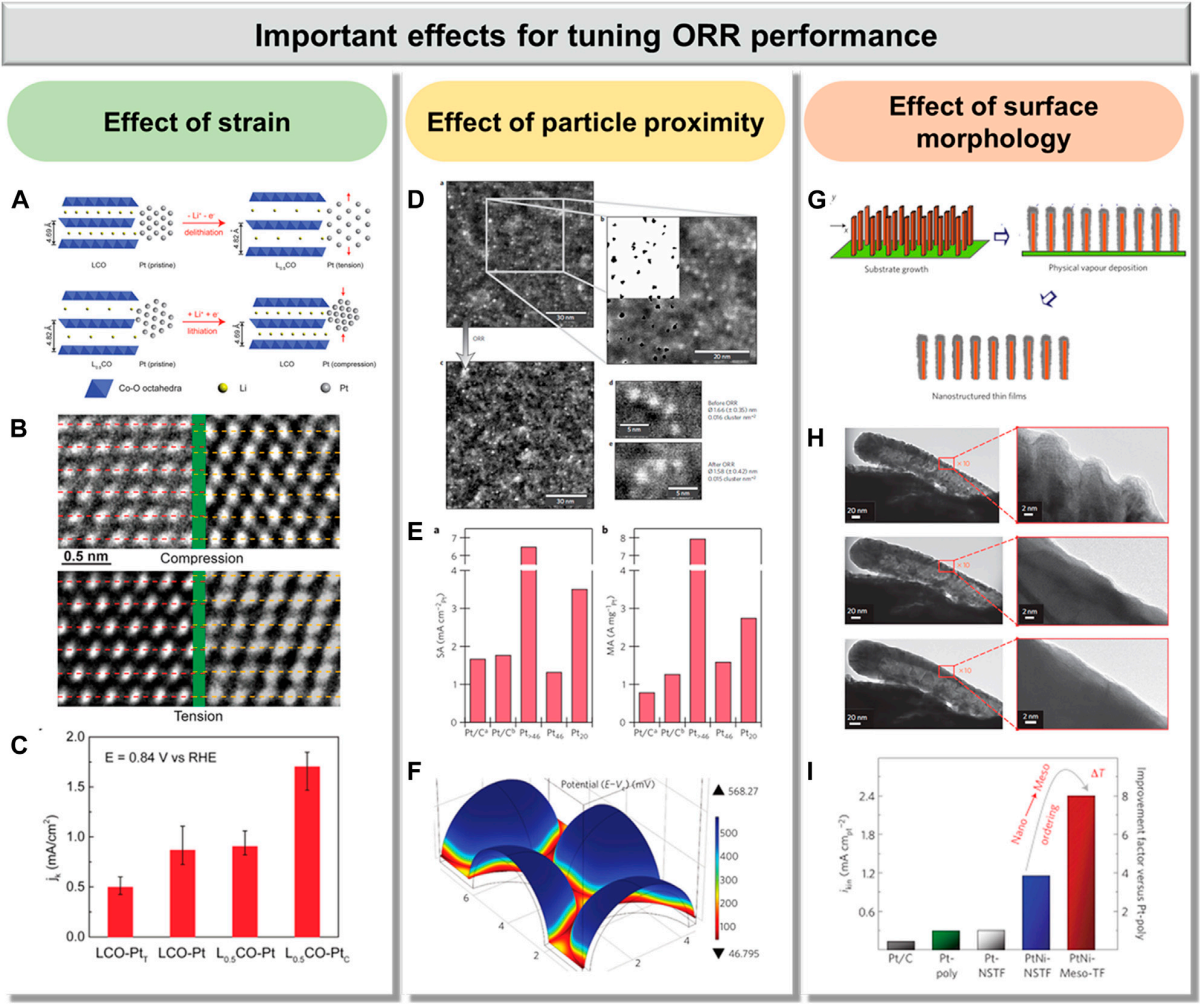
Emerging effects in Pt-based electrocatalysts for tuning ORR performance: **(A**–**C)** effect of strain, reproduced with permission from ref. 59; **(D**–**F)** effect of particle proximity, reproduced with permission from ref. 61, respectively; and **(G**–**I)** effect of surface morphology, reproduced with permission from ref. 67.

Few effective strategies to isolate and turn strain effects in electrocatalysis have been developed, because many fundamental effects are normally synchronously presented and affect the catalytic reaction. By selectively removing Cu atoms from a PtCu alloy, Strasser et al. successfully utilized the lattice strain for tuning the catalytic activity of dealloyed PtCu for ORR (Strasser et al., 2010). However, they are restricted to only investigating the effect of compressive strain because the lattice of Pt is larger than that of Cu. Subsequently, Cui et al. reported a strategy of using battery electrode material to directly control the lattice strain of Pt and thus adjust its ORR catalytic activity (Figure 2A) (Wang et al., 2016). During charging, the Li^+^ was extracted to form Li_0.5_CoO_2_ (Li_0.5_CO), resulting in the increasing of layer spacing from 4.69 to 4.83 Å. Thus, about 3% expansion in the substrate can cause uniaxial tension on Pt nanoparticles (LCO-Pt_T_). During discharge, the Li^+^ intercalated back into Li_0.5_CO and the lattice returned to its original spacing, generating compressive strain on Pt nanoparticles (L_0.5_CO-Pt_C_) (Figure 2B). As a result, compressive strain benefits the Pt with 90% enhancement in ORR activity, whereas tensile strain caused a negative effect with 40% suppression in activity (Figure 2C). Moreover, the effect of compressive strain can be maintained during the long-term durability test without apparent degradation.

Effect of particle proximity. In spite of the tremendous effort based on both experimental and computational modeling that has been dedicated to understanding the structure–performance relationship of the ORR activity on Pt-based nanostructures (Nie et al., 2015), it has not been fully understood yet. Especially, an understanding of the underlying difference between highly dispersed Pt nanoparticles and bulk Pt is still lacking. The studies concerning highly dispersed Pt-based catalysts are very limited because it is very hard to control and analyze the dispersion of Pt-based nanoparticles on high–surface area supports.

The influence of particle proximity in Pt-based nanostructures on ORR was fully investigated by Arenz et al. (Nesselberger et al., 2013). Other than traditional methods, the size and coverage of nanoclusters/nanoparticles can be independently changed using a laser ablation source to settle well-defined Pt nanoclusters/nanoparticles onto a glassy carbon support. Three different Pt nanoclusters with different sizes, {Pt_20_ [D (cluster diameter) = 0.6 nm], Pt_46_ (D = 0.8 nm), and Pt_>46_ (D = 2.3 nm)}, were investigated (Figure 2D). It is noted that Pt_>46_ with the largest size, rather than Pt_20_, displayed the highest mass activity because of the relatively small ECSA values of Pt_20_ (Figure 2E). Moreover, the number of adsorption sites has been found to be relatively low since Pt_20_ is very small (Figure 2F). Therefore, it is indicated that the mass activity of Pt electrocatalysts can be dramatically increased on well-defined Pt nanoclusters with optimal interparticle distance.

Effect of surface morphology. With the increasing development of Pt-based catalysts, in-depth basic and applied research has been focused on both surface and nanoscale systems (Bu et al., 2016b; Luo et al., 2018b). The main challenge is related to the possibility of synthesizing the unique surface structure of Pt-based materials (Zhao et al., 2019; Siddharth et al., 2020). A typical example is the formation of an order surface layer of Pt (111) skin by thermodynamically driven segregation of Pt from PtNi alloys *via* thermal annealing (Stamenkovic et al., 2007). Due to the modification of the electronic structure of the Pt (111) skin by the subsurface PtNi layer, this catalyst exhibited ultrahigh ORR activity of about two orders of magnitude higher than that of the commercial Pt/C catalyst. Thus, the exploitation of the Pt-skin structure in high–surface area catalysts may achieve the purpose of unprecedented ORR performance.

A class of Pt-based materials based on thin films with tunable surface morphology was reported by Stamenkovic et al. (van der Vliet et al., 2012). The PtNi nanostructured thin films (PtNi-NSTF) were first fabricated by depositing a PtNi thin film on an array of molecular solid whiskers (Figure 2G). Then, the PtNi mesostructured thin films (PtNi-Meso-TF) were prepared by thermal annealing of PtNi-NSTF in a reductive atmosphere of argon and hydrogen gases. Once the temperature reaches 300°C for 30 min, flatter and more ordered thin films with crystalline structures have been formed from the initial 3D surface morphology (Figure 2H). Due to the optimal near-surface composition and high crystalline surface morphology, PtNi-Meso-TF displayed an ultrahigh activity, which is 20 times greater than that of the commercial Pt/C catalyst (Figure 2I), indicating that the adjustment of surface morphology could alter the corresponding catalytic activity.

## Conclusions and Outlook

The high-performance Pt-based nanomaterials have been widely studied as highly active and stable electrocatalysts in order to realize the efficiency and commercial viability of fuel cell devices in the past decade. This minireview provides an overview of the recent development of high-performance Pt-based electrocatalysts for ORR. Salient examples of novel and preferred nanostructures, such as biaxially strained core–shell nanoplates, ultrafine jagged nanowires, nanocages with subnanometer-thick walls, and nanoframes with three-dimensional surfaces, are emphasized. The important effects on the atomic scale, including strain, particle proximity, and surface morphology, are discussed.

Recent years have witnessed rapid and significant progress in the synthesis of Pt-based electrocatalysts with superior ORR performance. However, the research studies in this field still face huge challenges for the practical applications of fuel cell devices, and many issues need to be addressed, such as the discrepancy between liquid half-cell and full-cell tests, the destruction of the structural integrity of these Pt-based materials, and the strategies for scale-up synthesis, including self-assembly, *in situ* calcination, and directed dealloying. To tackle these issues, the following research aspects are recommended: 1) *in situ* characterization techniques for investigating the catalytic active sites, 2) theoretical simulations for predicting the optimized morphologies/compositions, and 3) systematic investigation on the reason of the discrepancy between liquid half-cell and full-cell tests. Besides, the development of strong Pt-C interaction will also be of great significance for achieving high-performance Pt-based ORR electrocatalysts. Deeper insights into these significant breakthroughs in the developments of Pt-based materials will help identify the optimal conditions in order to achieve Pt-based electrocatalysts with high activity and stability toward ORR. There is no doubt that the commercialization of fuel cell devices will keep moving forward as continuous and rapid development of high-performance Pt-based ORR electrocatalysts takes place.
